# Anti-instinctive Learning Behavior Revealed by Locomotion-Triggered Mild Heat Stress in *Drosophila*

**DOI:** 10.3389/fnbeh.2020.00041

**Published:** 2020-04-07

**Authors:** Ruichen Sun, Joseph Delly, Emily Sereno, Sean Wong, Xinyu Chen, Yuxuan Wang, Yan Huang, Ralph J. Greenspan

**Affiliations:** ^1^Division of Biological Sciences, University of California, San Diego, La Jolla, CA, United States; ^2^Kavli Institute for Brain and Mind, University of California, San Diego, La Jolla, CA, United States

**Keywords:** *Drosophila*, learning, stress, operant conditioning, dopamine receptors

## Abstract

Anti-instinctive learning, an ability to modify an animal's innate behaviors in ways that go against one's innate tendency, can confer great evolutionary advantages to animals and enable them to better adapt to the changing environment. Yet, our understanding of anti-instinctive learning and its underlying mechanisms is still limited. In this work, we describe a new anti-instinctive learning behavior of fruit flies. This learning paradigm requires the fruit fly to respond to a recurring, aversive, mild heat stress by modifying its innate locomotion behavior. We found that experiencing movement-triggered mild heat stress repeatedly significantly reduced walking activity in wild type fruit flies, indicating that fruit flies are capable of anti-instinctive learning. We also report that such learning ability is reduced in dopamine 1-like receptor 1 (Dop1R1) null mutant and dopamine 2-like receptor (Dop2R) null mutant flies, suggesting that these two dopamine receptors are involved in mediating anti-instinctive learning in flies.

## 1. Introduction

The relationship between innate and learned behaviors has attracted a lot of attention since the mid-20th century (Tinbergen, 1951, 1963; Breland and Breland, 1961; Lorenz, 1991). Innate behaviors, also called instinct, are behaviors performed in their complete form the first time they were performed (Tinbergen, 1951). Innate behaviors have long been thought to be fixed and robust, and that learning does not seem to change them (Tinbergen, 1951, 1963). Recent studies on fruit flies, however, have shown that innate behaviors are fluid and can be modified by internal states, environmental cues, and learning, particularly operant learning (Suh et al., 2004; Turner and Ray, 2009; Taghert and Nitabach, 2012; Sengupta, 2013; Su and Wang, 2014; Wu et al., 2014; Baggett et al., 2018). The process of an animal associating certain behaviors of its own to a stimulus is operant learning (also called operant conditioning) (Skinner, 1963). Techniques of operant learning have been extensively used in training of animals and, sometimes, children (Gross, 2010). A classic operant conditioning example is B.F. Skinners' experiments on pigeons where a pigeon enclosed in a chamber received food pellets as rewards when it pecked a disc correctly (Ferster and Skinner, 1957). Based on this definition, the operant learning process modifies an animal's behavior. Not all behaviors, however, can be modified via operant conditioning. In the case of innate behavior-modifying learning, the learning that conforms to an animal's innate tendencies are easier to acquire than those that go against its innate behaviors (Seligman, 1970; Kandel et al., 2000). It is estimated that most of the operant behaviors studied in laboratory conditions are between the two extremes (instinct-conforming and instinct-opposing) mentioned (Seligman, 1970; Kandel et al., 2000). Instinct-opposing learning, which we term anti-instinctive learning, is one of the most challenging types of operant learning for an animal. Thus, studies on this type of learning have been limited.

Previous works on fruit fly courtship conditioning, in which a virgin male developed an unwillingness to engage in any courtship after being rejected repeatedly by a mated female, have indicated that flies may possess the capability for anti-instinctive learning (Siegel and Hall, 1979). However, researchers in this field have yet to agree on what specific signals were learned during the conditioning phase. The disagreement is largely due to the fact that conditioning a male fruit fly with a mated female would always involve a mixture of olfactory, visual, tactile, auditory, and gustatory cues (Tompkins and Hall, 1981; Tompkins et al., 1983; Ackerman and Siegel, 1986; Keleman et al., 2012; Joiner, et al.). Thus, a simpler behavior paradigm for anti-instinctive learning is called for.

Ideally, while still of a similar operant nature, an optimal anti-instinctive learning behavior paradigm should be a solitary one, with reliable and measurable behavioral changes in response to learning. In the fruit fly literature, a common behavioral indicator of learning is a fly's locomotion (regardless of the kinds of learning being studied), while heat has been extensively used as a stressor (Wustmann et al., 1996; Liu et al., 1999; Diegelmann et al., 2006; Ofstad et al., 2011; Yang et al., 2013; Baggett et al., 2018). One previous study showed that freely walking fruit flies are able to associate aversive heat with a specific location in an experimental chamber, which they avoid thereafter (Wustmann et al., 1996). Another study, using a similar behavioral setup as the one in the place learning study, has shown that fruit flies can develop learned helplessness when their normal locomotion is randomly being coupled with strong aversive heat stress (Yang et al., 2013). These studies hinted that a fruit fly's locomotion behavior itself can be used as an indicator of learning and that heat stress is a reliable stressor that flies find aversive. What remains unclear is whether a salient stressor by itself can make the flies learn to modify their robust walking behaviors. This illustrates the limited progress in our understanding of anti-instinctive learning in an animal model.

Leveraging the basic locomotion behavior of fruit flies as a behavioral model, with heat as a stressor, we designed a system called *LaserSync* to study anti-instinctive learning behavior of fruit flies. *LaserSync* is equipped with infrared laser emitters for fast heat delivery and with high-speed linear optical arrays for continuous location recording. Previous fruit fly behavioral apparatuses used Peltier elements or an electric board as a heating source (Wustmann et al., 1996; Diegelmann et al., 2006; Sitaraman et al., 2008; Yang et al., 2013; Batsching et al., 2016; Baggett et al., 2018). Compared to Peltier elements, which deliver heat stress to the animal by warming up the surrounding air, laser emitters increase the fly's body temperature directly while leaving the environment temperature unaffected, which may allow for more accurate body temperature control during heat delivery (Wustmann et al., 1996; Sitaraman et al., 2017). Compared to electric boards, which delivers heat stress to the animal only when the animal is in contact with the wires on the circuit board, laser emitters can deliver heat stress continuously to the fly (Batsching et al., 2016).

Using the *LaserSync* system, we first show that solitary fruit flies have robust incessant instinctive walking behavior in an experimental chamber. Then, we present that fruit flies possess anti-instinctive learning ability when exposed to recurring locomotion-triggered mild heat stress. Learning is evident both during and after the training phases, as flies receiving randomly occurring (not triggered by locomotion) mild heat stress consistently showed higher activity levels. Also, we report here that this anti-instinctive learning ability is reduced in flies lacking either the dopamine 1-like receptor 1 (Dop1R1) or dopamine 2-like receptors (Dop2R).

## 2. Materials and Methods

### 2.1. Fly Strains

The following fly lines were used: Canton-S (Greenspan lab stock), Dop1R1-Gal4 (Deng et al., 2019), Dop1R2-Gal4 (Deng et al., 2019), Dop2R-Gal4 (Deng et al., 2019), DopEcR-Gal4 (Deng et al., 2019), UAS-myr-EGFP (w[*]; Py[+t7.7] w[+mC]=10XUAS-IVS-myr::GFP^attP2^, Bloomington No. 32197).

### 2.2. Fly Husbandry

Adult virgin female and male flies of 2–7 days old were used in this study. Flies were reared in 23°C with 50–80% humidity and 12:12 light-dark cycles. All flies were assayed during the circadian time (CT) 0–5 and CT 7–11 (lights are turned on at CT 0 and turned off at CT 12). The fly food used in this study was made of dark corn syrup (30 mL/L), yeast (35 g/L), nipagin (1.125 g/L), propionic acid (7.5 mL/L), ampicillin (50 mg/L), chloramphenicol (50 mg/L), sucrose (15 g/L), and agar (10 g/L). All flies were isolated at eclosion and reared individually in 2.5 ml plastic isolation vials (Caplugs Cat. No.214-2002-010, Rancho Dominguez, California) containing 150 mg food. Isolating fruit flies at eclosion is to minimize the effect of social interactions in group rearing on flies' learning behavior. After behavioral experiments, all flies were returned to their original isolation vials and were kept until death.

### 2.3. LaserSync Setup

**Figure 1:**
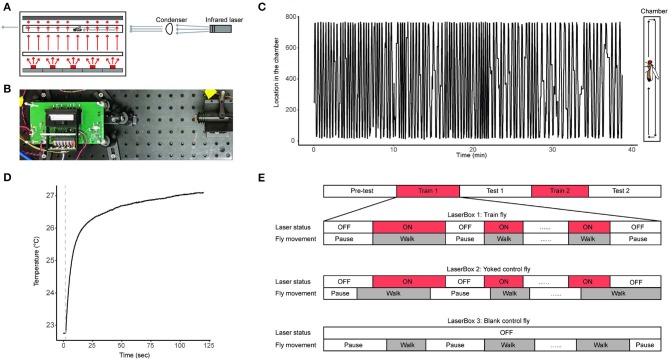
Behavioral experiment design. **(A)** A schematics of a LaserBox design along with an infrared laser emitter and a condenser. The large box shown on the left is the LaserBox, inside which are (from bottom to top) a red LED array, a diffuser, a glass tube with a fly in it, and a short-pass filter-covered position sensor. The red arrows illustrate light paths of the red lights from the LED array. The gray arrows represent light paths of the infrared light from the laser emitter. The LaserBox circuit board is not shown. **(B)** A photo of the actual LaserBox. The layout of the setup is identical to the schematics shown in panel A, except that the diffuser is positioned closer to the LaserBox than depicted in **(A)**, and that the position sensor is installed inside the black fixture, rendering it invisible in this picture. The green circuit board is the LaserBox circuit board not shown in **(A)**. **(C)** A 40-min recording of a fly's spontaneous activity in a LaserBox. The fly's spatial trajectory inside the tube is illustrated in the right diagram. **(D)** An example of a fly's body temperature profile when the fly is being irradiated by the laser emitter. The dotted line indicates when the laser emitter is turned on. The measurement of the fly's body temperature can be found in the Materials and Methods section. **(E)** Behavioral paradigm used in this study. The paradigm consists of 5 sessions: Pre-test, Train 1, Test 1, Train 2, and Test 2 sessions. The flies in the experimental group is called Train Flies, and flies in the two control groups are called Yoked control flies and Blank control flies. The timing relationship between a fly's walking activity and the status of the laser emitter in that fly's chamber is illustrated in the diagram, in which laser ON is labeled with red, and walking activity is labeled with gray.

We developed the *LaserSync* system for this study (Figures 1A,B, Table S1, Figures S1–S3). The *LaserSync* system consists of 4 LaserBoxes, an adapter board, a myRIO FPGA system, and an end-user computer. Each of the 4 LaserBoxes consists of a box fixture, an infrared laser emitter, and a LaserBox circuit board. Inside the box fixture are a 3D-printed fixture, a position sensor, a glass chamber, a diffuser, a custom-made 645 nm short-pass filter (dimension: 55 * 12.7 * 1 mm), a custom-made acrylic diffuser, and a red (630 nm) LED array. The glass behavioral chamber is a custom-made 48.7 * 4 * 2 mm^3^ transparent borosilicate tube with two custom made detachable 4 * 2 * 1 mm glass windows on both ends to provide complete enclosure. The glass chamber with its windows is held together in a custom-made 3D-printed acrylonitrile butadiene styrene holder, which the experimenter can quickly place the chamber in the LaserBox after allowing the fly voluntarily enters the chamber during experiment preparation. The 4 LaserBoxes are identical in design and can operate independently and simultaneously. In this study, each LaserBox accommodates one fly. A total of 4 flies can be assayed independently and simultaneously in each of the 4 LaserBoxes. A fly can receive heat stress from an infrared laser emitter situated at one end of the LaserBox while its locations inside the chamber of the LaserBox being constantly monitored by a linear optical sensor (Figures 1A,B). Via the adapter board, flies' trajectory information are transferred to the computer for storage. The control software is written in Laboratory Virtual Instrument Engineering Workbench (LabVIEW, propriety programming environment from National Instruments, Inc.). During each experiment, a fly's real-time location information along with the ON/OFF status of laser emitters is recorded by the LabVIEW software in technical data management streaming (TDMS) files (a binary file format developed by National Instruments, Inc.) (Figure 1C). The source code for the LaserSync system can be found here: https://github.com/Ruichensun/LaserSync.

We use infrared laser emitters (wavelength: 808 nm) for heat delivery. The infrared light beam emitted from the laser emitter is collimated via a condenser before reaching the fly inside the chamber. The laser emitter can warm up the fly's body temperature from room temperature to up to 45°C (Figure S4). In our study, we chose 26°C to 27°C (about 5 − 6°C above room temperature) as the temperature range for training the fruit flies, and we call this the mild heat stress treatment. The mild heat stress' effect on the body temperature of a fly is validated by measuring body temperature when the fly is being irradiated by the infrared light. To do so, one inserts a thermocouple data acquisition module-connected mini hypodermic probe (OMEGA Engineering, Inc., Cat. No. TC-08 and No.HYP1-30-1/2-T-G-60-SMP-M) in an adult fly's abdomen and placing the fly in the center of the glass chamber irradiated with the infrared light. The fly's body temperature can be readily measured as long as the probe is inside the fly's body (Figure 1D). This invasive temperature measurement is not conducted during behavior experiments. The laser emitters' ON/OFF status is controlled by an experimenter operating the software system installed on the end-user computer. Of the 4 LaserBoxes, one has an external monochromatic camera [FLIR Integrated Imaging Solutions, Inc., Cat. No.Flea3 1.3 MP Mono USB3 Vision (e2v EV76C560)] positioned at the top of the box fixture. This camera, together with the linear optical sensor, provides visual information for experimenters to deliver prompt heat stress to the fly during experiments.

### 2.4. Behavioral Experiment

We designed a 5-session behavioral protocol to study the anti-instinctive learning behavior in flies (Figure 1E). The 5 sessions are: Pre-test, Train 1, Test 1, Train 2, and Test 2 sessions. Each of the 3 Test sessions (Pre-Test, Test 1, and Test 2) are 10 min long and no heat stress was given during these sessions. Each of the 2 Train sessions (Train 1 and Train 2) consists of up to 20 episodes of mild heat stress treatments which are only given to the fly when it moves. The mild heat stress treatment stops when the fly stops walking. If the fly has received 20 episodes of mild heat stress treatments, or if it has been stationary for 8 min, the Train session concludes and the experiment moves on to the next session. The cutoff at the 8-min was chosen because (1) different flies need different amount of heat stress to finish the 2 Train sessions, and not setting a cutoff time would result in the total duration of an experiment vary greatly from fly to fly; and (2) a fly's prolonged inactivity during the Train sessions in itself is an indicator of it having learned to inhibit its walking activity. In addition, to prevent flies staying at the edges of the chamber during Train sessions, the Train flies receive mild heat stress treatment when it is at either end of the glass chamber, the ends defined as the left and right most 3 mm segment of the chamber (Soibam et al., 2012).

Two types of controls are used: yoked control and blank control. Train, Yoked control, and Blank control flies were assayed simultaneously in separate LaserBoxes. During Train sessions, Train fly's movement triggers the laser emitter of its own LaserBox to release heat stress as well as the laser emitter in the yoked control fly's LaserBox. This means that the yoked control fly receives identical mild heat stress treatment to that of the Train fly, regardless of whether the yoked control fly is moving or not. The blank control fly does not receive any mild heat stress treatment throughout the entire experiment. Flies were randomly assigned to any of the Train, Yoked control, and Blank control groups at the beginning of each experiment.

### 2.5. Data Curation

During experiments, flies' raw behavioral trace data was stored by our custom-written LabVIEW programs in the TDMS file format (National Instruments, Inc., Austin TX., the United States). After each experiment, the TDMS files are converted into comma-separated value (CSV) format using custom-written MATLAB script (The MathWorks, Inc., Natick, MA, the United States). Subsequent data analysis and data visualizations are done in R, a programming language and a free software environment for statistical analysis (The Comprehensive R Archive Network). In addition to raw trace data, attributes of each fly such as eclosion dates and gender are recorded in a separate CSV file as a reference to match each fly's basic attributes with its behavioral trace data.

The trace data quality control protocol is as follows: (1) flies inactive more than 90% of the time during the Pre-test session are excluded from the data set, as a lack of robust baseline walking behavior before Train sessions indicates the fly's physiological condition potentially deviating from healthy baseline; (2) Data with incorrect or missing attributes, or errors during experimental procedures, are also removed from the dataset.

### 2.6. Statistical Analysis

The Kruskal-Wallis test followed by pairwise Wilcoxon rank sum tests is used for all statistical comparisons unless otherwise noted. Confidence intervals are calculated using permutation tests with 10,000 permutations of the raw data. Statistical significance is shown as: ^*^(*p* < 0.05), ^**^(*p* < 0.01), ^***^(*p* < 0.001), ^****^(*p* < 0.0001); n.s., not significant (*p* > 0.05). Statistical analysis is performed using R.

### 2.7. Immunohistochemistry and Confocal Imaging

The brains of adult progenies from crossing UAS-myr-EGFP^attP2^ and the four DopR mutant Gal4s were used for confocal imaging. Adult fly brains are dissected following a previously described protocol (Wu and Luo, 2006). The dissected brains are stained according to the Janelia Farm Research Campus' FlyLight IHC-Anti-GFP protocol (https://www.janelia.org/project-team/flylight/protocols) with modifications. Specifically, the dissected brains were first fixed using 2% paraformaldehyde in phosphate buffered saline (PBS) for 55 min at room temperature (RT) while nutating. Then, the brains were washed with 0.5% Triton X-100 diluted in PBS (PBT) for 4 × 10 min while nutating. After post-fix washes, we used 5% goat serum diluted in PBT for 1.5 h of blocking while nutating. After blocking, the brains were stained with mouse nc82 primary antibody (33.3 μL/mL, Developmental Studies Hybridoma Bank, University of Iowa, Iowa City, IA) for 36–48 h, with the first 4 h at RT while nutating, and the remaining time at 4°C while nutating. After the primary antibody incuation, the brains were washed using 0.5% PBT for 4 × 30 min while nutating. Next, the brains were incubated with secondary antibody, Alexa Fluor 568 Goat anti-Mouse (2.5 μL/mL, Life Technologies Corporation, Carlsbad, CA), diluted in 5% goat serum in PBT for 72 h, with the first 4 h at RT while nutating and the remaining time at 4°C while nutating. After the secondary antibody staining, the brains were washed using 0.5% PBT for 4 × 30 min while nutating. After the washes, the brains were ready for imaging. We did not stain GFP using anti-GFP antibody. Instead, we took advantage of the fluorescent signals from constitutively expressed GFP (green fluorescent protein) in the fly brain. Brains were imaged immediately using ZEISS LSM 800 with Airyscan system with dual color channels (488 and 561 nm). The maximum intensity projection of all confocal Z-stacked images are presented in this study.

## 3. Results

### 3.1. The Train Flies Reduce Activity During the Train Sessions

**Figure 2:**
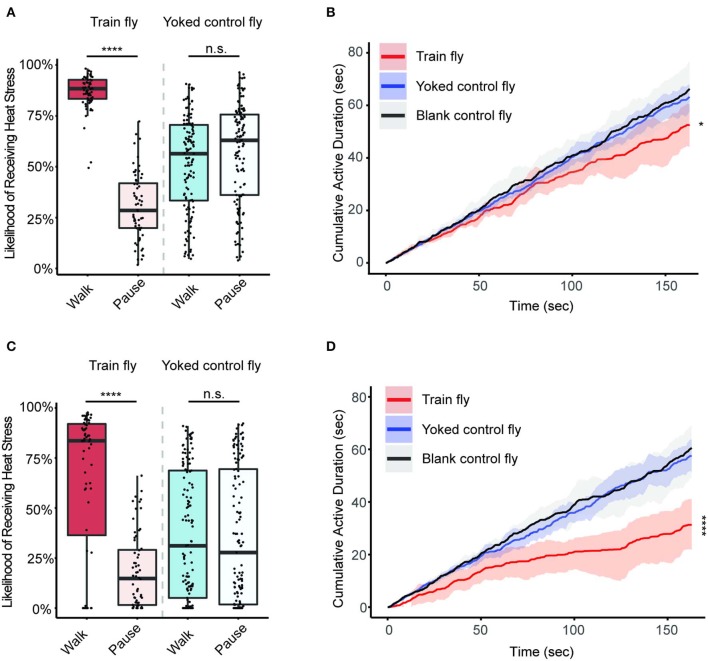
The train flies reduce activity during the train sessions. Sample size: 64 (Train flies), 125 (Yoked control flies), 72 (Blank control flies). **(A)** Likelihood of receiving heat stress when a fly is at different behavioral states during Train 1 session. The bright red box corresponds to the likelihood of Train flies receiving heat stress during walking. The light red box corresponds to the likelihood of Train flies receiving heat stress during pause. The bright blue box represents the likelihood of Yoked control flies receiving heat stress during walking. The light blue box represents the likelihood of Yoked control flies receiving heat stress during pause. **(B)** Flies' cumulative active duration (CAD) during Train 1 session. Data from the Train flies is shown in red, while the Yoked control fly data and the Blank control fly data are shown in blue and gray, respectively. The solid lines represent the median in each group, and the shaded regions correspond to the confidence intervals. The CAD of Train flies at the end of Train 1 session (the 163th sec) is significantly lower than the CAD of other two groups (*p* < 0.05, Kruskal-Wallis test followed by pairwise Wilcoxon rank sum tests) **(C)** Likelihood of receiving heat stress when a fly is at different behavioral states during Train 2 session. The color reference is identical to that of **(A)**. **(D)** CAD during Train 2 session. The color reference is identical to that of **(B)**. The CAD of Train flies at the end of Train 2 session (the 163th sec) is significantly lower than the CAD of other two groups (*p* < 0.0001, Kruskal-Wallis test followed by pairwise Wilcoxon rank sum tests). ****p < 0.0001.

To understand whether flies are able to learn anti-instinctively, we first assess the nature of the mild heat stress experienced by different groups of flies (Figure 2). We compared the likelihood of receiving mild heat stress when a fly is in different behavioral states (walking or pause). The likelihood of receiving mild heat stress during one behavioral state is the fraction of time duration a fly receives mild heat stress when it is at that behavioral state.

During the Train 1 session, the Train flies receive mild heat stress 88% ± 2% of the time when they walk, which is significantly different from the likelihood of receiving mild heat stress when they are not walking (28% ± 7%, *p* < 0.0001) (Figure 2A). During the same Train 1 session, however, Yoked control flies receive mild heat stress with comparable likelihood during both walking and resting: 56% ± 6% during walking, and 63% ± 6% during resting (*p* > 0.05), indicating that their walking behaviors are not preferentially punished as those of the training flies are (Figure 2A). The reasons why the Train flies do not receive mild heat stress 100% are (1) the Train flies receive the mild heat stress when they stay at the ends of the chamber, and (2) the mild heat stressors are manually controlled by an experimenter observing the Train flies movement, and if a Train fly showed walking for longer than half a second, the laser emitter (source of the mild heat stressor) will be turned on.

In the Train 2 session, the Train flies' likelihood of receiving mild heat stress during walking and resting continue to differ significantly: Train flies experience mild heat stress 84% ± 7% of the time during walking and 15% ± 8% during resting (*p* < 0.0001) (Figure 2C). It is worth noting that a subset of the Train flies showed complete lack of activity during the Train 2 session, and therefore these flies' likelihood of receiving mild heat stress is 0, contributing to the wide confidence intervals. In contrast to the Train flies, during the same Train 2 session, Yoked control flies experience comparable likelihood of receiving mild heat stress when they are walking or in pause. During walking, Yoked control flies' median likelihood of receiving mild heat stress is 31% ± 17%. When staying still, a Yoked control fly's likelihood of receiving mild heat stress is 28% ± 17% (*p* > 0.05) (Figure 2C). This indicates that the mild heat stress the Yoked control flies receive are random.

If flies are capable of anti-instinctive learning, the Train flies, which experience heat stress during walking and not during pauses, would show less movement during the Train 1 or Train 2 sessions compared to either the Yoked control flies or the Blank control flies. To test this hypothesis, we measured cumulative active duration (CAD) of all flies during the two Train sessions (Figure 2). The time each fly takes to complete one Train session varies from fly to fly due to the operant nature of the experiment. As a result, the minimum length of both Train sessions, 163 s, was used for comparing the CAD across different groups of flies and different sessions. This time point is referred to as the end of Train 1 and Train 2 sessions.

At the end of Train 1 session, the CAD of Train flies (CAD: 52.6 ± 8.0 s) is significantly smaller than that of the Yoked control flies (CAD: 63.1 ± 5.2 s, *p* < 0.05) and the Blank control flies (CAD: 66.3 ± 10.6 s, *p* < 0.01) (Figure 2B). At the end of the Train 2 session, the Train flies move significantly less (CAD: 31.3 ± 9.7 s) compared to those in the Yoked control flies (CAD: 57.5 ± 6.1 s, *p* < 0.0001) or the blank flies (CAD: 60.6 ± 10.0 s, *p* < 0.0001) (Figure 2D). These results indicate that during the Train sessions, the Train flies have gradually learned to walk less compared to flies in the two control groups, a sign of learning.

### 3.2. Train Flies' Continue to Show Less Active After Training Ends

**Figure 3:**
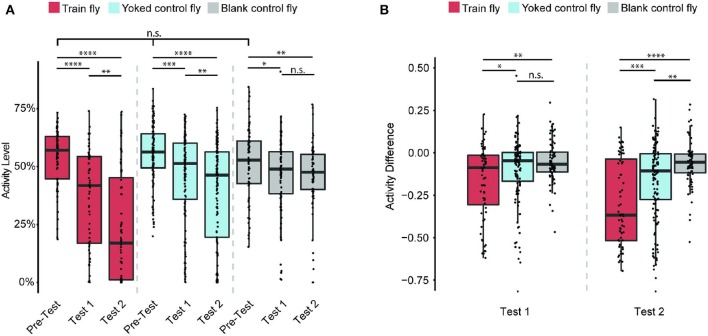
Train flies' continue to show less activity after training ends. Sample size: 64 (Train flies), 125 (Yoked control flies), 72 (Blank control flies). **(A)** Train, Yoked control, and Blank control flies' activity levels (percentage of time a fly is active during the entire test session) in Pre-Test, Test 1 and Test 2 sessions. During Pre-Test session, all three groups of flies show comparable activity levels during Pre-Test. During each subsequent test session (Test 1 and Test 2), Train flies and Yoked control flies activity levels decreased significantly (Kruskal-Wallis test followed by pairwise Wilcoxon rank sum tests). **(B)** All three groups of flies' activity difference (AD, the change of activity level shown in A) of Test 1 and Test 2 from that of Pre-Test (i.e., the AD of Test 1 is the activity level of Test 1 minus the activity level of Test 2. To avoid confusion, here we do not use the percentage as the unit). The AD showed that the activity level of Train flies decreases significantly more than that of the two control groups (Kruskal-Wallis test followed by pairwise Wilcoxon rank sum tests). *p < 0.05, **p < 0.01, ***p < 0.001, ****p < 0.0001.

Does the flies' learned behavior observed in both Train 1 and Train 2 sessions persist after each Train session ends? To answer this question, we measured each fly's activity level in Pre-Test, Test 1, Test 2 sessions (Figure 3A). The activity level is defined as the percentage of time the fly is active during the entire duration of the session.

During the Pre-Test session, Train flies activity levels are 57% ± 4%. After Train 1 session, these flies activity level decreased to 42% ± 7% (*p* < 0.0001). After Train 2 session, their activity levels drop to 17% ± 10% (*p* < 0.0001). The significant decrease of activity level after each Train session indicates that the anti-instinctive learning effect continues beyond training. For the Yoked control flies, their initial activity levels are 56% ± 2%, and moderate decreases in activity were observed after each Train session: 51% ± 3% in Test 1 (*p* < 0.001), and 46% ± 5% in Test 2 (*p* < 0.0001). The Blank control flies' activity levels were: 53% ± 5% in Pre-Test; 49% ± 3% in Test 1 (p < 0.05); and 48% ± 3% in Test 2 (p < 0.01). As all three groups of flies show a decrease from Pre-Test to Test 2 sessions in activity levels, it seems that the activity level decrease is larger in Train flies compared to the control groups. To understand the size of the learning effect, we measured the change of activity level (also called the activity difference, AD), which is the difference in activity levels between either Test session (Test 1 or Test 2) and the Pre-Test session (Figure 3B). For example, if a fly's activity level during Pre-Test session is 70% and its activity level during Test 1 is 40%, its AD of Test 1 is 40–70%, which is −30%. Since AD's unit is a percentage, but it is an actual change in activity level, not a percent change of activity, here we use only the numeric value of AD (−0.3), and do not use the percentage as the unit. A decrease in activity levels before and after Train sessions indicates the presence of anti-instinctive learning effects. After Train 1 session, the Train flies' AD is −0.09 ± 0.08, while the Yoked control flies' AD is −0.05 ± 0.02 (*p* < 0.05), and the Blank control flies' AD is −0.07 ± 0.03 (*p* < 0.01). This indicates that after Train 1, the activity changes in Train flies are already significantly different from the activity changes observed in the control groups. The difference is more pronounced after Train 2 session: the Train flies' AD are −0.37 ± 0.11, while the Yoked control flies' AD are −0.11 ± 0.04 (*p* < 0.001) and the Blank control flies' AD are −0.06 ± 0.02 (*p* < 0.0001). This result suggests that the learned locomotor inhibition observed during Train sessions persists after the training ends, further confirming that the flies are capable of anti-instinctive learning.

### 3.3. Yoked Control Flies Show a Moderate Decrease in Activity Level

**Figure 4:**
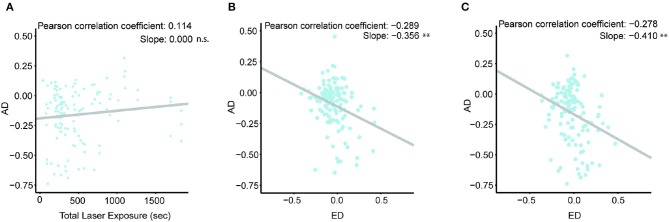
Yoked control flies show a moderate decrease in activity level. Sample size: 125. Correlation coefficient, slope, and the statistical significance are shown on the top right corner. **(A)** Correlation between AD and total laser exposure. **(B)** Correlation between AD and ED after Train 1 session. **(C)** Correlation between AD and ED after All Train sessions. **p < 0.01.

The AD results revealed an interesting phenomenon: some Yoked control flies' activity levels show a larger decrease than the rest of the Yoked control flies after 2 Train sessions. Given the operant nature of the assay, this phenomenon raises a question: what are the factors underlying the observed reduced activity levels in these Yoked control flies? Two factors are possible: the effect due to prolonged heat stress exposure and the effect of anti-instinctive learning. To find out if prolonged heat stress exposure is affecting Yoked control flies walking behavior, we analyzed the correlation between each Yoked control fly's AD (between Pre-Test and Test 2) and its total duration of heat stress exposure during the two Train sessions (Figure 4A). If exposure to mild heat stress in itself has a cumulative effect on the flies, the longer a fly experiences mild heat stress, the greater a change its activity level will be. Our linear analysis results showed that the Yoked control flies' AD does not show a significant correlation with their total mild heat stress exposure (*p* > 0.05). This suggests that increasing mild heat stress exposure to a Yoked control fly does not significantly change its activity level. Moreover, all three groups of the flies assayed in this experiment lived similar amount of days (Figure S5). Taken together, we have not observed significant cumulative effects, such as exhaustion or helplessness, of random mild heat stress exposure in the Yoked flies in our experiments.

Our second hypothesis, for the reduced activity levels in subsets of Yoked control flies, is that some Yoked control flies may have learned anti-instinctively to inhibit their own walking during the Train sessions if they experience mild heat stress more often during walking than during pause. During the experiment, when Yoked control flies experience mild heat stress is determined by their respective Train fly counterparts. This experience may not be equally random for every Yoked control fly. By chance, it is possible that a subset of yoked control flies may have received more heat stress during walking compared to resting. Therefore, to understand if being exposed to more heat stress when a Yoked control fly is walking than when it is in pause affects the fly's AD, we analyzed the correlation between the randomness of heat stress exposure and AD. The randomness of heat stress exposure is defined as the exposure differential (ED), which is the difference between the likelihood of receiving heat stress during walking and the likelihood of receiving heat stress during pause (Equation 1). As an example, if a Yoked control fly experiences heat stress 50% of the time during walking and 50% of the time during the pause, its' ED will be 50–50%, which is 0; if another yoked control fly experiences heat stress 80% of the time during walking, and 30% of the time during the pause, its ED would be 80–30%, which is 0.5.

(1) $$\begin{array}{l} \text{Exposure Differential}=\text{P}\left(\text{heated}|\text{walking}\right)-\text{P}\left(\text{heated}|\text{pause}\right) \end{array}$$

Our analysis shows that the Yoked control flies' ED in Train 1 session has a significant negative correlation with their AD between Pre-Test and Test 1 (*p* < 0.01) (Figure 4B). This indicates that the subset of Yoked control flies that experience heat stress more often during walking may have learned to reduce their activity levels. Furthermore, our results show that the Yoked control flies' ED in the entire experiment showed a similarly significant negative correlation with their AD before and after the 2 Train sessions (*p* < 0.01) (Figure 4C). This result, together with previous results, further validates the flies' ability to perform anti-instinctive learning when they experience more heat stress during walking than during pause.

### 3.4. Dop1R1 and Dop2R Are Involved in Flies' Anti-instinctive Learning

**Figure 5:**
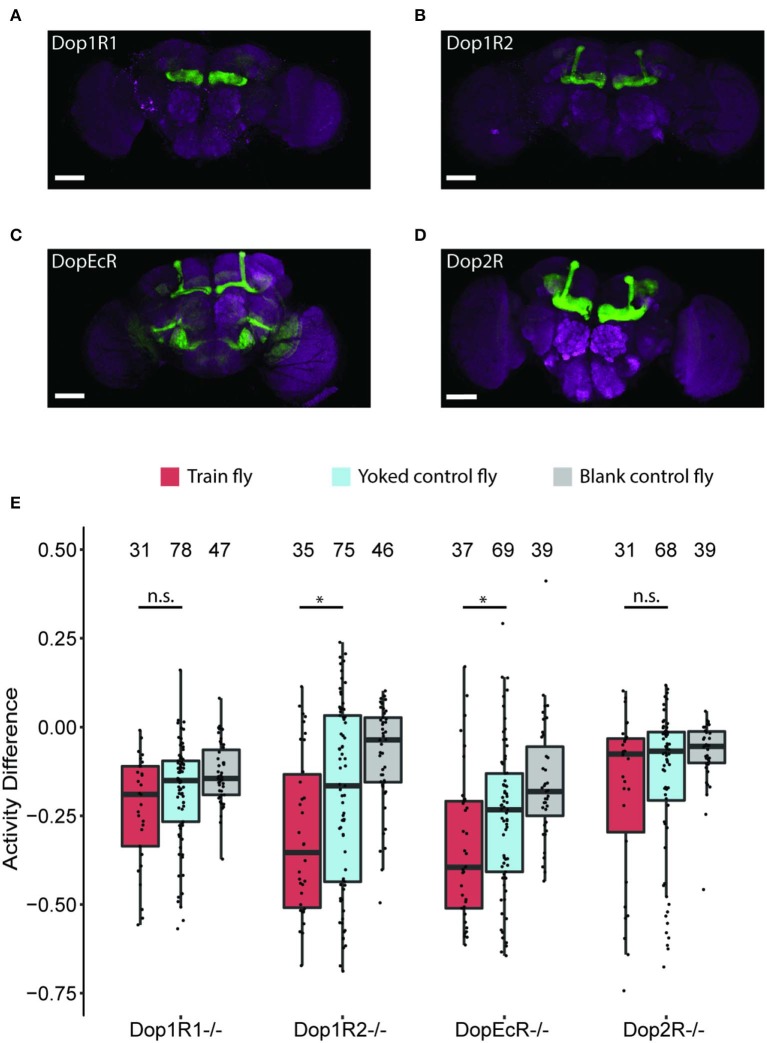
Anti-instinctive learning performance in dopamine receptor null mutants. **(A–D)** The expression patterns of the dopamine receptors (anterior view, scale bar: 100 μm). **(A)** Dop1R1. **(B)** Dop1R2. **(C)** DopEcR. **(D)** Dop2R. **(E)** Anti-instinctive learning performance in dopamine receptor null mutants. Dop1R1 and Dop2R are involved in flies' anti-instinctive learning revealed by the activity difference (AD) between Pre-Test and Test 2. Sample sizes are indicated at the top of the graph. *p < 0.05.

Dopamine is an evolutionarily conserved neurotransmitter involved in the control of motor behaviors (Kass-Simon and Pierobon, 2007; Barron et al., 2010). In higher organisms, dopamine has been reported to be associated with behaviors such as reward-seeking, executive control, mood regulation, and learning (Willner, 1983; Schultz, 2001; Packard and Knowlton, 2002; Balleine et al., 2009). The dopamine signaling pathway is highly conserved between the fruit fly's brain and mammals. Fruit flies also employ dopamine for a variety of behaviors, including learning (Van Swinderen and Andretic, 2011; Berry et al., 2012; Waddell, 2013; Yamamoto and Seto, 2014; Sitaraman et al., 2015). Four types of dopamine receptors are found in the fly's brain: dopamine 1-like receptor 1 (Dop1R1), dopamine 1-like receptor 2 (Dop1R2), dopamine 2-like receptor (Dop2R), and dopamine/ecdysteroid receptor (DopEcR) (Hauser et al., 2006). All 4 types of receptors are expressed in the mushroom bodies (Deng et al., 2019). Previous studies showed that Dop1R1 is involved in aversive and appetitive olfactory learning, arousal level regulation, innate and startle-induced motor control, and temperature preference behaviors (Kim et al., 2007; Han et al., 2008; Lebestky et al., 2009; Kong et al., 2010; Bang et al., 2011; Pitmon et al., 2016; Sun et al., 2018). Dop1R2 plays a role in olfactory memory formation and courtship drive (Berry et al., 2012; Zhang et al., 2016). Dop2R has been reported to be important in memory formation and olfactory learning (Draper et al., 2007; Qi and Lee, 2014; Scholz-Kornehl and Schwärzel, 2016). Lastly, DopEcR modulates memories of courtship conditioning and sensitization to ethanol (Ishimoto et al., 2013; Petruccelli et al., 2016; Hinojos et al., 2017). Given the ample evidence of the importance of dopamine receptors in learning, it is worth exploring the role of dopamine receptors in anti-instinctive learning. To do so, we tested the activity level changes of dopamine receptor null mutants. These dopamine receptor-null mutants have previously been reported and were generated by replacing either the first coding exon (Dop1R1, Dop1R2, DopEcR) or the last seven common exons (Dop2R) with Gal4 sequence, which is a yeast transcription activator (Deng et al., 2019) (Figures 5A–D, Figure S6).

We measured the post-Train 2 session AD of each of the 4 dopamine receptor null mutant lines (Figure 5). Our result shows that the ADs of Dop1R1 and Dop2R mutant Train flies are −0.19 ± 0.08 and −0.08 ± 0.09, which are not statistically different from their Yoked control counterparts (Dop1R1 Yoked control flies: −0.15 ± 0.03, *p* >0.05; Dop2R Yoked control flies: −0.07 ± 0.04, *p* > 0.05). In contrast, the ADs of Dop1R2 and DopEcR mutant Train flies are −0.35 ±0.14 and −0.40 ± 0.01, which are significantly different from their respective Yoked control flies (Dop1R2 Yoked control flies: −0.17 ± 0.12, *p* < 0.05; DopEcR Yoked control flies: −0.23 ± 0.05, *p* < 0.05). Reduced activity changes in Dop1R1 and Dop2R null mutant suggests that these two receptors are involved in the fly's anti-instinctive learning process.

## 4. Discussion

Since the discovery of courtship conditioning, no other types of anti-instinctive learning behavior in fruit flies has been reported. Using a custom-designed laser-based behavioral system, we report a new anti-instinctive learning behavior of fruit flies. Flies are subjected to recurring mild heat stress during 2 Train sessions, and the flies' activity levels are measured before, during, and after each Train session. Our results showed that fruit flies are capable of reducing their activity levels when (and after) their walking activity triggers mild heat stress, a sign of anti-instinctive learning. Previous behavioral studies on freely moving fruit flies frequently used ≥ 37°C temperature as stressor in order to induce a strong learning outcome from flies (Brembs, 2003; Diegelmann et al., 2006; Yang et al., 2013; Bath et al., 2014; Baggett et al., 2018). Using ≥ 37°C temperature as stressors, however, may cause physiological damage to the fly or even kill the fly within a few seconds of exposure. Our result shows that flies are able to learn with mild heat stress of around 27°C. Being able to induce learning in flies with a milder heat stressor is desirable in studies such as ours when prolonged exposure to the stressor is needed.

The learning effect observed in Train flies is further reinforced when compared to the Yoked control flies. Due to the operant nature of the experiments, some Yoked control flies also received more heat stress when they are walking than when they are in pause. This subset of Yoked control flies subsequently showed a larger reduction in their post-training activity level. This suggests that the stronger the mild heat stress correlates with walking (compared to with inactivity), the greater the anti-instinctive outcome. Also, we have not observed in the Yoked control flies a significantly cumulative effect, such as exhaustion or helplessness, of the mild heat stress used in the study. Previous studies of learned helplessness behavior were conducted in significantly different ways compared to our study: such as using a much stronger temperature (37°C), a different heat delivery sequence (the Train flies were heat-stressed only during inactivity, instead of during walking), or a different type of stressor (electric grid) (Yang et al., 2013; Batsching et al., 2016).

Our results also showed that Dop1R1 or Dop2R null mutant Train flies' activity changes after two Train sessions are not significantly different from their Yoked control group counterparts. This indicates that lacking either of these two dopamine receptors reduces the flies' anti-instinctive learning. In contrast, Dop1R2 and DopEcR null mutant Train flies show significantly more negative AD compared to their Yoked control group counterparts, a pattern similar to that of the wild type CS flies. All 4 types of dopamine receptors have been reported to show strong expression in the mushroom bodies of the fly brain (Deng et al., 2019). The mushroom bodies (MB), known for their crucial role in associative memory in fruit flies, are a pair of mushroom-shaped neuropils located at the center of the fly's brain (Heisenberg et al., 1985; de Belle and Heisenberg, 1994; McGuire et al., 2001; Aso et al., 2014). Each of the two MBs is comprised of Kenyon cells whose axons form three distinct lobes within MB: α/β, α′/β′, and γ lobes (Ito et al., 1997; Crittenden et al., 1998; Aso et al., 2014). While all three lobes have been reported to be involved in associative memory formation (Heisenberg et al., 1985; McGuire et al., 2001; Pascual and Préat, 2001; Aso et al., 2014; Kirkhart and Scott, 2015; Yamagata et al., 2015; Kim et al., 2017), a recent study reported that Dop1R1, and not Dop1R2, expressed in the intrinsic MB Kenyon cells was required for the inhibitory effects of dopamine neurons on startle-induced locomotion (Sun et al., 2018). In light of our results and the literature, it would be interesting to see where in the MB do Dop1R1 and Dop2R play a role in the anti-instinctive learning process.

## Data Availability Statement

The raw data supporting the conclusions of this article will be made available by the authors, without undue reservation, to any qualified researcher.

## Author Contributions

RS and RG conceived and designed the project. RS designed and built the hardware and software of behavioral setup. RS, JD, ES, SW, XC, YW, and YH contributed to data collection. RS analyzed and interpreted the data. RS and RG drafted and revised the manuscript.

### Conflict of Interest

The authors declare that the research was conducted in the absence of any commercial or financial relationships that could be construed as a potential conflict of interest.
