# Supplementary material for: Anti-instinctive Learning Behavior Revealed by Locomotion-Triggered Mild Heat Stress in Drosophila
Source: Front Behav Neurosci. 2020 Apr 7;14:41. doi: 10.3389/fnbeh.2020.00041 (PMC7179688; doi:10.3389/fnbeh.2020.00041)
Supplement: Supplementary file 1 [file Data_Sheet_1.PDF]

Supplementary Material

Table S1. A List of LaserSync Hardware's Main Components

Item	Catalog Number	Source
Linear optical sensor	TSL1406R	AMS-TAOS USA Inc., Plano, TX
808nm laser emitter	RLDB808-120-3	Roithner Lasertechnik GmbH, Vienna, Austria
645nm short-pass filter	SP645-R55X12.7mm	Midwest Optical System, Inc., Palatine, IL
Condenser	ACL12708U	Thorlabs Inc., Newton, NJ
myRIO-1950	782690-01	National Instruments Inc., Austin, TX
Acrylic diffuser	8505K754	McMaster-Carr, Elmhurst, IL
Glass chamber	BRT-2-4-50	Friedrich & Dimmock Inc., Millville, NJ
Glass window	12-550-100	Fisher Scientific, Hampton, NH

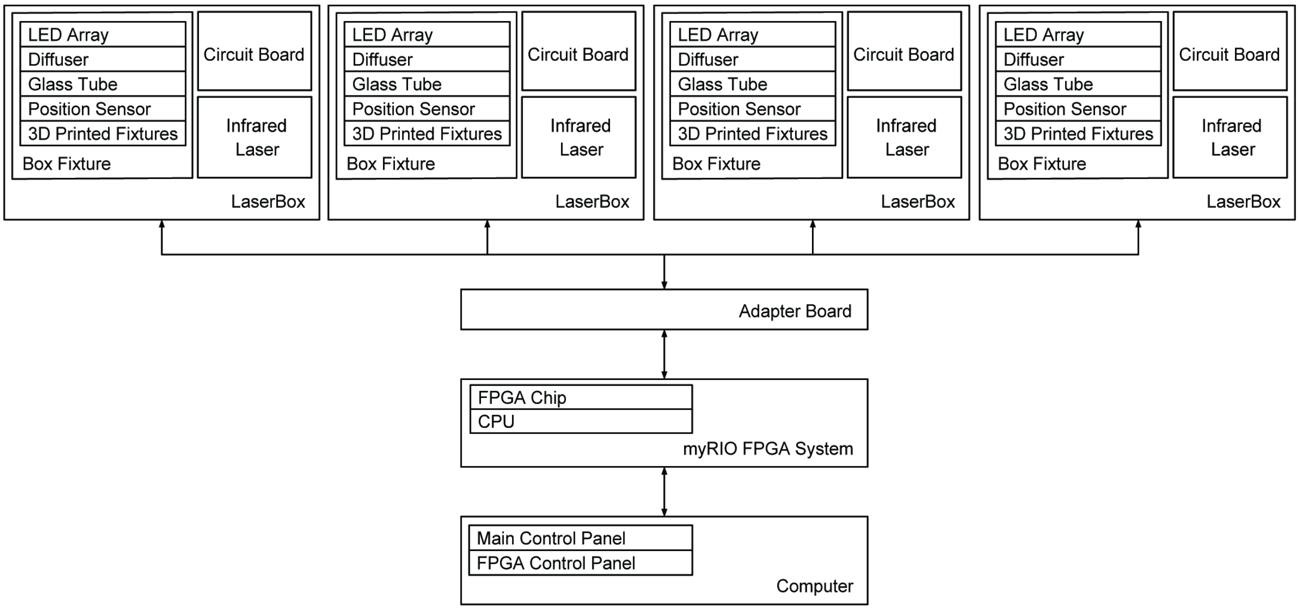

Figure S1: The design architecture of the LaserSync system

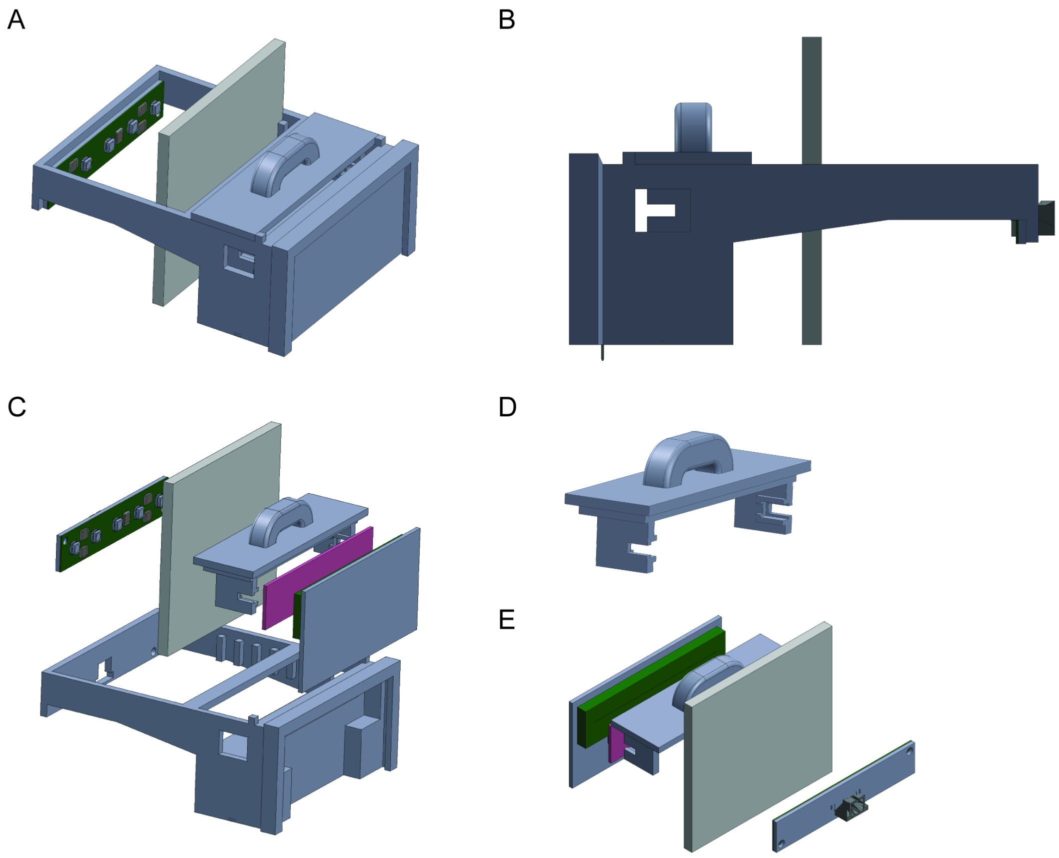

Figure S2: The schematic of a LaserBox. **(A-B)** The side view of a LaserBox. **(C)** The exploded view of a LaserBox with fixture. From left to right (top): red LED array, diffuser, glass tube holder, short-pass filter, position sensor, and the cover of the back of the fixture. Underneath these parts is the fixture. **(D)** The holder. **(E)** The exploded view of a LaserBox without the fixture.

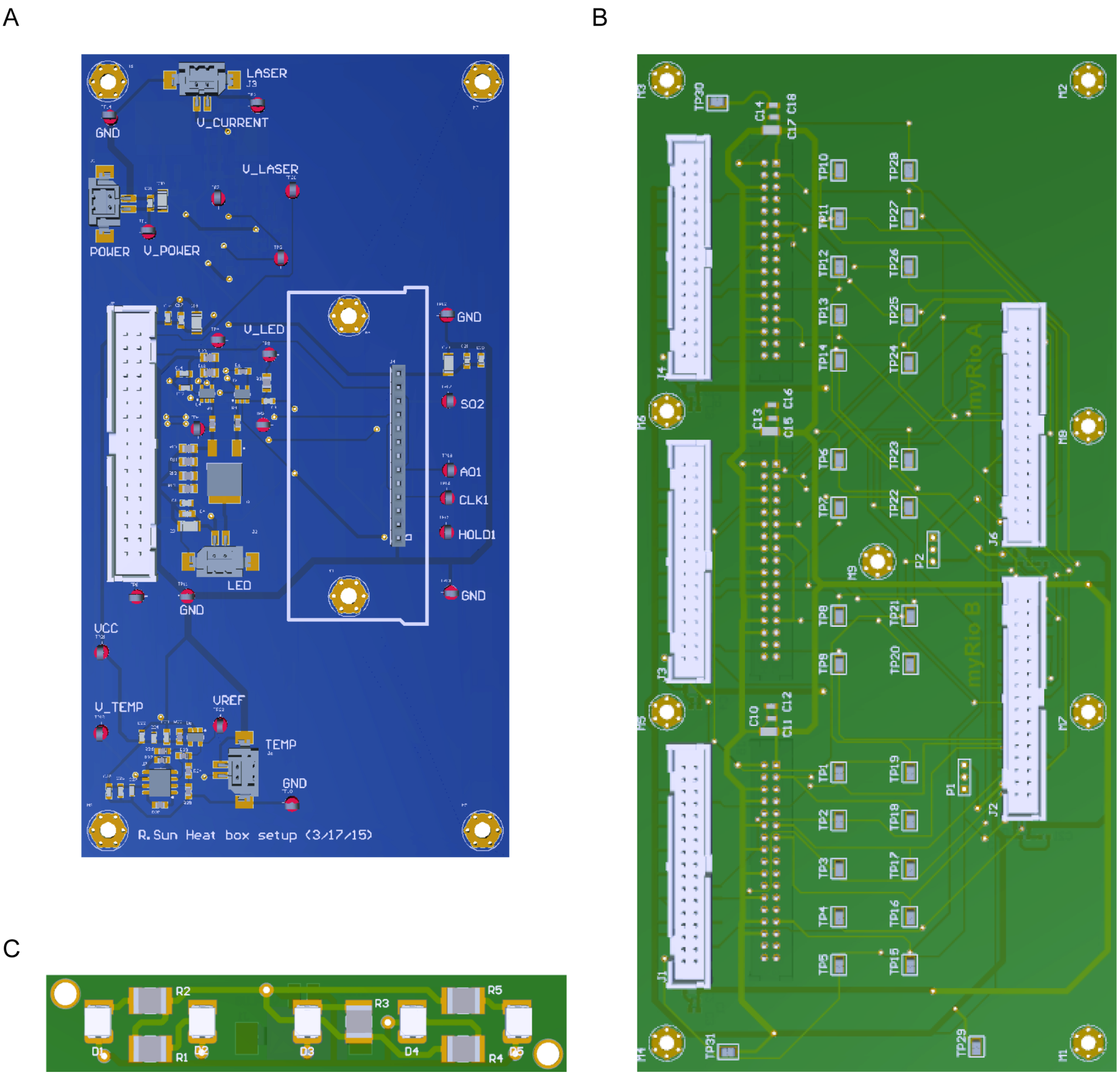

Figure S3: The schematic of circuit boards of the LaserBox. **(A)** The LasesrBox's circuit board, **(B)** The adapter board, **(C)** The LED panel circuit board.

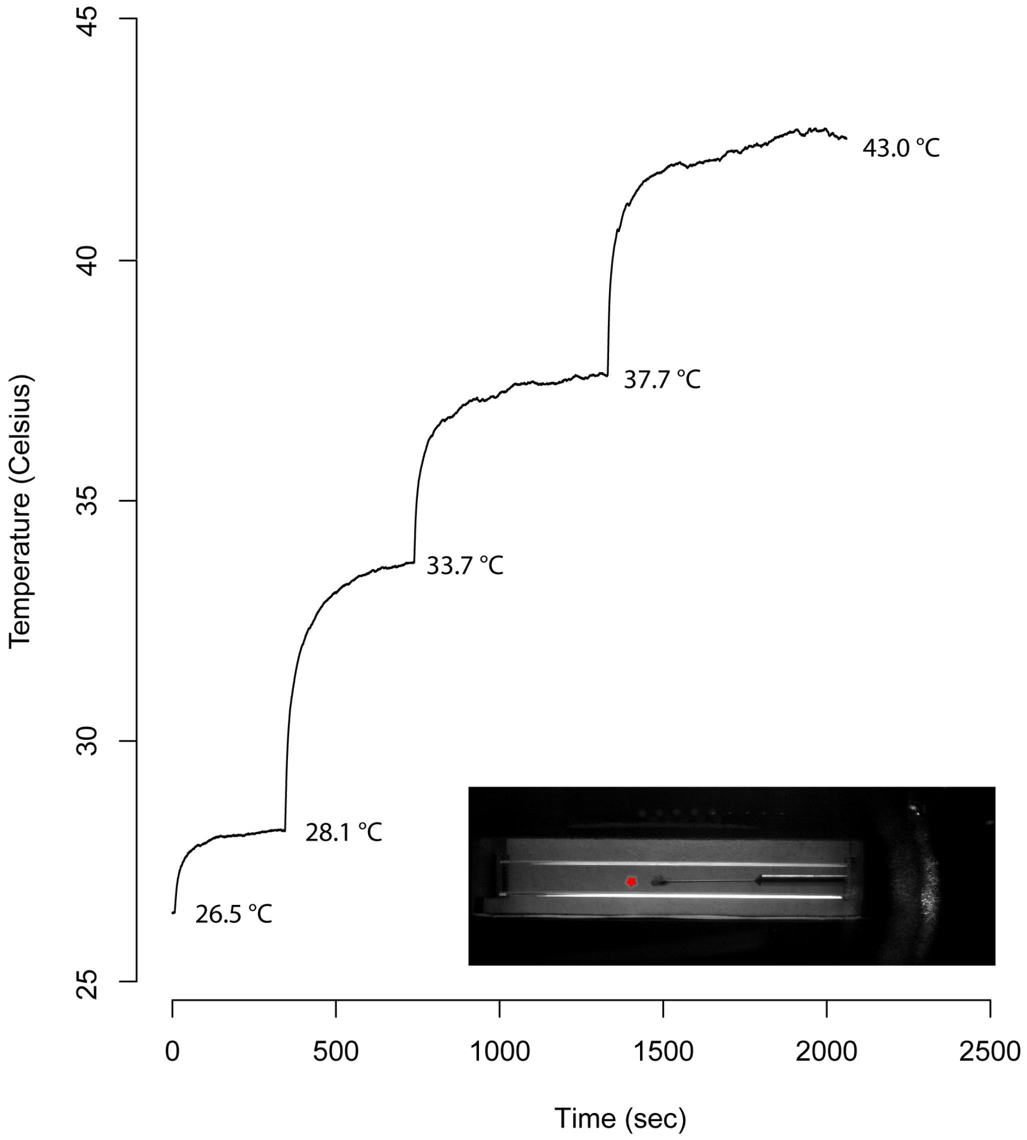

Figure S4: The laser emitter in the LaserBox is capable of warming up the fly temperature from room temperature to $43°C$. The inset is an immobilized fly with its temperature being measured. The temperature increase is achieved by increasing electric currents going through the laser emitter.

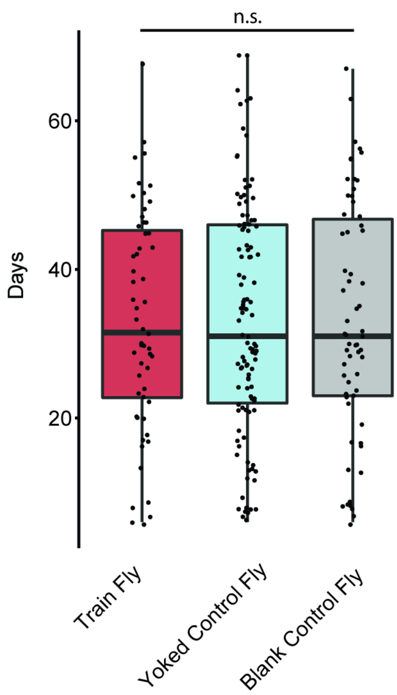

Figure S5: The total number of days Train flies, Yoked control flies, and Blank control flies lived after they had gone through the behavioral experiment. No statistical significance was observed among the three groups. Sample size: 64 (Train flies), 125 (Yoked control flies), 72 (Blank control flies).

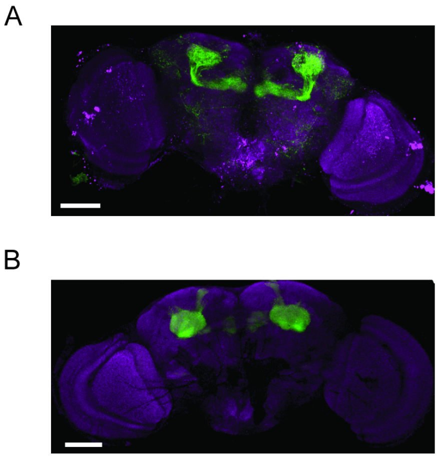

Figure S6: The expression patterns of dopamine receptor Dop1R1 and Dop2R (posterior view, scale bar: $100\,\mu m$). **(A)** Dop1R1. **(B)** Dop2R.
